# The Sixth Year Molar

**Published:** 1888-09

**Authors:** W. H. Hall

**Affiliations:** Martins Ferry, O.


ARTICLE V.

### THE SIXTH YEAR MOLAR.

BY W. H. HALL, D. D. S., MARTINS FERRY, O.

[Read before the Ohio Valley Dental Society, held at Steubenville, Ohio, July 2nd, 1888.]

This, the first permanent tooth to appear through the gum, "is not unlike the temporary teeth in that it has its origin in and from the primitive dental groove," (Garretson,) where the temporary teeth are germinated. About the fourth month of fœtal life the papilla, or germ of the sixth year molar, appears; eruption taking place about that period in life indicated by its name. On account of its early development and eruption it is generally neglected, its relation to the permanent set being not understood.

Dr. Woolley compares this tooth to a waif, or outcast in the street, neglected through ignorance of its value. To the dentist the points of difference between it and the temporary teeth are readily distinguished, but we often hear the patient say, "that is one of my *first* teeth," which we readily grant, but it is nevertheless a member of the permanent set and if lost will not be replaced. This latter statement often causes great surprise to fond parents who would be unwilling to bring to the altar of sacrifice a *permanent* tooth. Just at this point the patient should be given a practical lesson in dentistry, and until we have popular education on care of the teeth, we must do missionary work on our individual responsibility, improving every opportunity. Because little or nothing is done for the sixth year molar before necessity compels professional attention, we are often puzzled to know what to do—extract it or not extract it, that is the question. It is absurd to say a general rule covers the ground. The circumstances vary with the age of the patient, the structural condition and arrangement of the remaining teeth, all aid us in making an intelligent and wise disposition.

Dr. Andrieu, of Paris, in a paper on the above subject, read before the Dental Section of the International Medical Congress, at Washington recently, held that "the sixth year molar being in development, eruption and structure, an organ of transmission from the temporary set to the permanent, it should be extracted when the permanent teeth are in position. More room is gained thereby and a longer period of usefulness insured to those remaining." This is French, you know, imported, but I am willing to believe extremists do not always follow their own doctrines. Granting the two points concerning development and eruption, we will note one point, viz.: *structure*, and show that the permanent molars cannot be classed with the temporary teeth. Even in external appearance they give evidence of being more dense and more enduring in structure, but it is proven by analysis that the permanent teeth contain a larger amount of inorganic material and a less proportion of organic than the temporary teeth.

Prof. Frank Abbott said he could see no difference in structure between the sixth year molar and the other permanent teeth under the microscope, but a very marked difference was apparent between that and the temporary teeth. Wedl makes the following report of examinations of specimens of four deciduous and ten permanent teeth as to density: "In the former the density varied from 1.00 to 2.17; in the permanent teeth the variation was between 1.98 and 2.53. The difference between their extremes being .89 and .36 in favor of the permanent teeth.

At one time it was the practice to save all the first permanent molars, but we are not willing to accept that doctrine unqualified. I will consider briefly three different stages only, when we may be called upon to decide the destiny of this tooth. First, that period just prior to the eruption of the bicuspids; second, after the eruption of the second bicuspids and just prior to the eruption of the second molar; third, after the complete eruption of the second molar. The importance of preserving the sixth year molar till the first period is passed cannot be questioned. Extraction before this time would be a serious disturbance to the progress of the development of both bicuspids and an interference with the regular formation of the alveoli and symmetrical outlines of the maxilla. The second stage demands study and the exercise of our best judgment. If the molar can be saved for years of future usefulness it should be filled, but if the other teeth give evidence of poor structure, its period of usefulness does not extend beyond the eruption of the second molar. It should be extracted to allow the second molar to move forward and occupy the space, thus affording more room for the third molar which will consequently be a better tooth. There is danger of the bicuspids separating, falling back and even turning partly around, thus destroying the articulation if the molar is extracted too soon. In the third stage, preserve the tooth if possible, extract under protest. If extracted a large masticating surface is lost, perhaps in a double sense, because the second molar will tilt forward, thus presenting only the posterior edge or a corner to articulate, leaving practically no masticating surface. The sixth year molar sometimes appears as a puzzling factor in cases of irregularity; however, I would not hesitate between a sound bicuspid and a decayed molar, to extract the bicuspid, provided the molar could be saved. With its broad masticating surface, the molar is of more value than the bicuspid, and would save much derangement of the articulation in regulating. Finally, we must preach and teach, to enlighten our patients concerning the true relation and worth of the sixth year molar; and our further duty is to operate on this tooth by filling, or with forceps tempered with wisdom and good judgment.—*Exchange.*

